# The p53-p21 pathway inhibits ferroptosis during metabolic stress

**DOI:** 10.18632/oncotarget.25362

**Published:** 2018-05-15

**Authors:** Amy Tarangelo, Scott Dixon

**Affiliations:** Scott Dixon: Department of Biology, Stanford University, Stanford, CA, USA

**Keywords:** ferroptosis, p53, p21, ROS, glutathione, Autophagy

The p53 tumor suppressor protein is mutated or functionally inactivated in approximately 50% of human cancers. Historically, p53 was thought to suppress tumorigenesis by initiating apoptosis, cell cycle arrest, and senescence. Yet recent work suggests that each of these functions is dispensable and the mystery of how p53 suppresses tumor formation remains unsolved [3].

Mutations in p53 can be used to understand the function of this protein in controlling different biological processes. One interesting p53 variant with three lysine to arginine mutations (i.e. p53-3KR) retains the ability to suppress tumor formation, despite lacking the ability to induce apoptosis, cell cycle arrest or senescence. An exciting report in 2015 suggested that p53-3KR suppressed tumor formation by disrupting the uptake of cystine - the disulfide form of the thiol-containing amino acid cysteine - and inducing an iron-dependent form of non-apoptotic cell death termed ferroptosis [2]. Cystine/cysteine are needed for the synthesis of the antioxidant tripeptide glutathione, and loss of cystine uptake therefore results in glutathione depletion, iron-dependent ROS accumulation, and ferroptotic cell death. This model therefore links the regulation of metabolism by p53 to tumor suppression via the induction of non-apoptotic cell death.

A question left largely open from previous work was how the expression of the fully wild-type p53 protein affected ferroptosis sensitivity. In our recent study, we asked how increasing the expression of wild-type p53 impacted ferroptosis sensitivity in human and mouse cancer cells [7]. To our surprise, increased expression of wild-type p53 consistently suppressed ferroptosis in response to cystine deprivation. While the degree of ferroptosis suppression varied between cell lines, wild-type p53 stabilization was never observed to sensitize to cystine deprivation-induced death. We subsequently found that transactivation of the canonical p53 target gene *CDKN1A*, encoding p21^CIP1/WAF1^, was essential for wild-type p53 to suppress ferroptosis. Notably, the p53-3KR mutation used in prior studies cannot transactivate *CDKN1A*, possibly explaining why different results were obtained with wild-type p53 versus the p53-3KR mutant.

**Figure 1:**
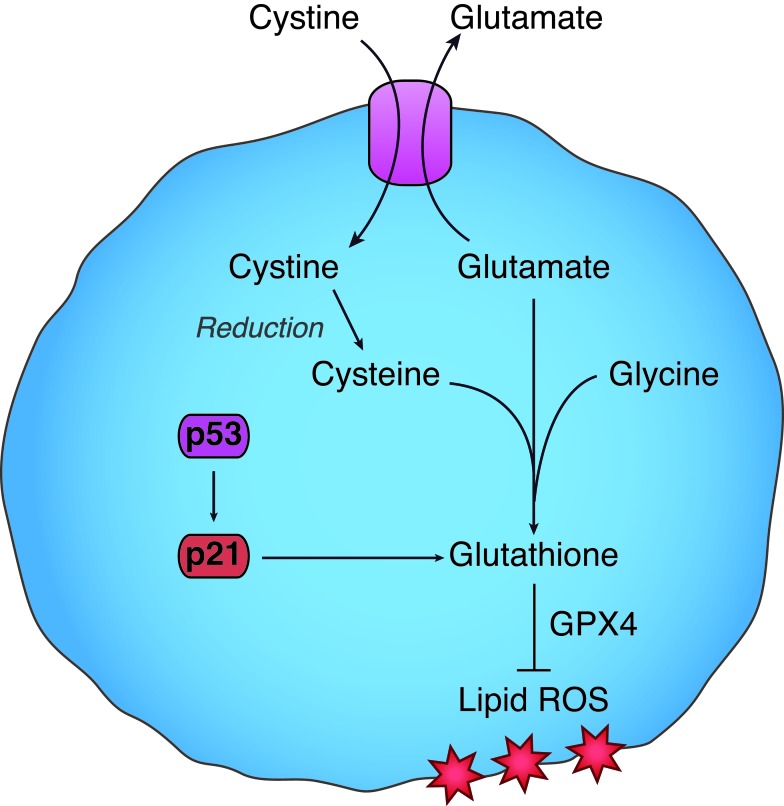
The p53-p21 pathway suppresses ferroptosis induced by cystine deprivation Ferroptosis is an oxidative, non-apoptotic form of cell death that can be triggered by deprivation of cystine. Cystine is imported into the cell where it is reduced to the amino acid cysteine. Cysteine is necessary for the synthesis of the tripeptide antioxidant, glutathione, which is used by the lipid hydroperoxidase GPX4 to suppress the accumulation of lipid reactive oxygen species (ROS, red stars). Stabilization of p53 leads to increased expression of p21^CIP1/WAF1^ (p21). p21 promotes the conservation of glutathione during ferroptosis to suppress the formation of lipid ROS and prolong cell survival.

Mechanistically, activation of the p53-p21 pathway most likely inhibits ferroptosis by suppressing the accumulation of toxic lipid ROS and promoting the conservation of the cysteine-derived antioxidant, glutathione [7] (Figure 1). These findings are broadly consistent with a prior study showing that the p53-p21 pathway promotes cancer cell survival in response to serine deprivation by enhancing glutathione levels and maintaining redox balance [4]. Precisely how p21 promotes glutathione synthesis or conservation is not clear from either study. One possibility is that p21 helps recycle oxidized glutathione to reduced glutathione. Alternatively, p21 could decrease the export of glutathione from the cell. A third possibility is that p21 expression reduces the consumption of glutathione. Independent of p53, p21 is known to regulate cell survival, metabolism, and oxidative stress responses [5], and our new results provide an additional impetus to scrutinize the connection between p21 and intracellular metabolism.

Our work establishes one mechanism by which the p53-p21 pathway can prevent ferroptosis in existing cancer cells in the face of cystine deprivation. These results do not rule out the possibility that p53 can promote ferroptosis in incipient tumor cells *in vivo*, or that p53 can promote ferroptosis under other conditions. It might seem paradoxical that a canonical tumor suppressor like p53 would promote the survival of cancer cells. However, many human cancers retain the ability to express wild-type p53, p53-mediated pro-survival effects have been observed previously in other cancer contexts [1] and few mutations of *CDKN1A* are observed in any cancer type. Thus, one intriguing possibility is that the need to adapt to elevated levels of oxidative stress and limited glutathione *in vivo* [6] favors the retention of a functional p53-p21 pathway in certain cancers. Testing this hypothesis *in vivo* in appropriate genetically-engineered models will be an important goal of future studies.
